# Counteracting Traditional Knowledge Erosion: An Ethnobotanical Survey in Valle Imagna (Bergamo, Italy) to Foster Intergenerational Transfer

**DOI:** 10.3390/plants14223477

**Published:** 2025-11-14

**Authors:** Fabrizia Milani, Martina Bottoni, Alessia Maiellaro, Alfonso Crisci, Piero Bruschi, Claudia Giuliani, Gelsomina Fico

**Affiliations:** 1 Department of Pharmaceutical Sciences, University of Milan, Via Luigi Mangiagalli 25, 20133 Milan, Italy; martina.bottoni@unimi.it (M.B.); alessia.maiellaro@gmail.com (A.M.); claudia.giuliani@unimi.it (C.G.); gelsomina.fico@unimi.it (G.F.); 2 Botanical Garden G.E. Ghirardi, Department of Pharmaceutical Sciences, University of Milan, Via Religione 25, 25088 Toscolano Maderno, Brescia, Italy; 3 Institute of BioEconomy, National Research Council (CNR), Via Madonna del Piano 10, 50019 Sesto Fiorentino, Florence, Italy; alfonso.crisci@cnr.it; 4 Department of Agricultural, Environmental, Food and Forestry Science and Technology, University of Florence, Piazzale delle Cascine 18, 50144 Florence, Italy; piero.bruschi@unifi.it

**Keywords:** Valle Imagna, ethnobotanical survey, children, schools, parents, grandparents, TEK erosion

## Abstract

Although younger generations are not always given a prominent role in ethnobotanical surveys, studying intergenerational knowledge transfer should still be a primary interest, in the context of traditions’ erosion, globalization, disinterest and plant blindness. Our study was designed to describe the situation of knowledge transfer and to find potential solutions to counteract erosion by involving the children. This ethnobotanical survey involved students from primary and secondary schools of Valle Imagna (Bergamo, Italy) through different meetings and structured questionnaires to record their traditional knowledge on medicinal plants. The children were then asked to become an active part of the project by interviewing their families. All data recorded were archived in a database for statistical analysis. Students (number = 112) reported 41 plant species, with 36% reporting at least 3 species each. Forty percent of their use reports were related to exotic species or purchased plant material. The most reported species were *Matricaria chamomilla* L. and *Camellia sinensis* (L.) Kuntze with common preparations such as infusions from commercial products. Parents (n = 96) reported 76 species and grandparents (n = 35) 52. Statistical analysis showed correlation between traditional knowledge and age/gender, with older generations and female gender correlated to deeper knowledge. Our results suggest deep erosion and a clear lack of intergenerational knowledge transfer. However, our project serves as evidence of the concrete role ethnobotany holds in safeguarding the remaining cultural heritage of a territory, fostering preservation from the outset with the participation of younger generations.

## 1. Introduction

The involvement of the younger generations, specifically of children, during ethnobotanical surveys can still be considered unusual [1].

On one hand, however, some authors have demonstrated that this population group should be considered as a separate group worthy of being studied in itself, because of it owning a body of knowledge on plant species even potentially distinct from the one of the adults [1]. While it is clearly rare to find papers in the literature in which children are shown to have deeper knowledge than adults [1], by leaving behind this section of the population precious information could be undervalued and regrettably lost.

On the other hand, the erosion of Local and Traditional Ecological Knowledge (LEK and TEK) and the difficulty in their intergenerational transmission is still a hot topic of discussion in ethnobotany and has been highlighted by many authors throughout the years [2,3]. In fact, younger generations typically seem to retain only limited portions of TEK from the elderly of a community especially, but not exclusively, in developed countries [3,4,5,6]. This hardship in the transmission of knowledge could be explained by different factors, such as socio-economic, cultural and environmental. Additionally, urbanization and globalization, though with some rare exceptions, are considered majorly responsible for cultural homogenization and the erosion of TEK [7]. Although grandparents still have an important role in familial management, spending a lot of time with grandchildren while both parents are at work, the moments spent together in outdoors activities, specifically in the fields and woods, are rarer and rarer. Especially in more urbanized areas, grandparents themselves do not always have the knowledge to pass down to the younger generation anymore, nor the necessity to go out of their way and gather wild plants for the support of the family. On top of that, if not properly stimulated, children are likely to become less and less outdoorsy, as well as less interested in plants and their surrounding environment and, in recent years, this phenomenon seems to have become more and more prominent even in rural territories [3,8]. Other than being common sense, scientific reports have shown that encouraging children to participate in outdoor activities or to study medicinal or edible plants (perceived as ‘useful’) is not only mentally and physically beneficial but can also foster their acquisition of LEK [9] and fight plant blindness [10]. Other authors also highlighted the role of active participation in scientific research in improving learning and attention to the research topic [11].

In a previous investigation of ours, conducted throughout the territory of upper Valle Imagna (province of Bergamo, Northern Italy), we recognized patterns of erosion of traditional knowledge already from within the older generations [12]. However, no children had been involved in that research. Thus, we chose to conduct a side ethnobotanical investigation in the same territory, by actively involving first children of the schools of Valle Imagna, then their families. This study was designed with the aim of taking a snapshot of the situation concerning intergenerational knowledge transfer and knowledge erosion, while finding potential solutions to counteract this erosion and reverse the trend, by fostering generational exchange and encouraging children to attentively observe the surrounding environment and to give traditional knowledge its due.

## 2. Results and Discussion

### 2.1. Total Pool of Active Respondents

Following the collection of the consent forms and the compiled questionnaires, the active participants in the project were:

Primary school (1st, 2nd and 3rd grade): n = 111 (no questionnaires)
Primary school (4th, 5th grade): n = 48
Lower secondary school (6th, 7th grade): n = 64
Parents’ generation: n = 96 (age 28–57)
Grandparents’ generation: n = 35 (age 57–90)
The total number was n = 354 respondents, of which n = 243 through questionnaires.

### 2.2. Primary School—1st, 2nd and 3rd Grade (N. of Participants = 111)

The uses reported by the younger children by means of free listing and group chat belonged to the medicinal, food and ritual sectors. Because this project focused specifically on medicinal plants, we present the primary data concerning the medicinal sector of use.

Although the children were aware of multi-ingredient plant-based commercial preparations used by their parents and grandparents, they reported several local and traditional medicinal uses concerning spontaneous plants of the territory, cultivated plants and single herb commercial products (i.e., tea leaves). We focused specifically on these.

The informants reported a total of 16 plant species, belonging to 14 botanical families.

Leaves of *Aloe vera* (L.) Burm.f. (commonly cultivated in the valley), *Castanea sativa* Mill., *Morus alba* L. and *Robinia pseudoacacia* L. were all reported for skin diseases and traumas, used as wound healing or as natural plasters. Satchels filled with seeds of *Prunus avium* L. were described as useful to treat burns, applied directly on the skin after refrigeration. In the same category of skin traumas, *Allium sativum* L. and *Mentha* spp. were reported as useful remedies against insect bites and stings. Specifically, an informant told us in great detail how his old neighbor once rubbed a clove of garlic (bulb) on the child’s leg after they had been stung by a wasp. Another informant reported that their grandmother would rub mint leaves from their garden every time the child was bitten by insects.

*Mentha* spp. or *Camellia sinensis* (L.) Kuntze (commercial) leaves were used in infusion for digestive problems, such as stomachache. The same use was reported for the fresh squeezed juice of *Citrus* x *limon* (L.) Osbeck, especially drunk in warm water. The bulbs of *Allium cepa* L. were also reported as a remedy against stomach and tummy ache. An informant actually told us they learnt from their grandparents that slices of fresh onion externally applied on the tummy and kept in place with a damp cloth could be used against digestive problems (“It really helps!”).

*A. cepa* was also known as a remedy against upper airways problems (i.e., colds): either the vapors from slices of the bulbs are inhaled to open the airways or a half bulb is kept on the bedside table for the entire night. Steam inhalations of an infusion of *Rosmarinus officinalis* L. leaves or *Matricaria chamomilla* L. (often commercial) flowerheads were considered useful to treat the same problems. A homemade syrup prepared with flowers of *Tilia cordata* Mill. was reported as a remedy against cough.

The infusion of *M. chamomilla* flowerheads was also considered a relaxant remedy to induce sleep.

The bulbs of *A. cepa* were also used in the category musculoskeletal problems, specifically applied sliced on contusions.

For the category cardiovascular problems, nettles (*Urtica dioica* L.) were reported as beneficial for circulation (“When you get stung by nettles, you’d think it is just hurtful, but they actually do you some good!”; “Indeed! Because it is good for your circulation! My mum and granny told me so”).

Concerning the category eye problems, one student learnt from their dad that slices of *Solanum tuberosum* L. tubers, placed on the eyes, can help mitigate redness and inflammation.

Finally, concerning the category general condition, the children all agreed that fresh squeezed juice from sweet orange (*Citrus* × *aurantium* L.) and lemon (*C. limon*) can help a person stay healthy if drunk every day.

All of these remedies had been learnt in the family environment, taught to them especially by the grandparents.

However young these children were, we were astounded by the depth of traditional knowledge retained by this group. Other than the medicinal uses, these younger children showed an emotional and affective bond to the plants around them, not only being able to draw them in a surprisingly realistic and accurate way but also giving us reports such as “When I’m feeling down, I always hug a beech tree” (*Fagus sylvatica* L.) or “Look! This pinecone is my lucky charm; I always keep it with me”.

Because of their young age, we did not plan to involve this group in interviewing their parents and grandparents via structured questionnaires. However, they took it upon themselves to participate in the project as homework and also through chats with their families, drawings and school assignments to learn more about some of the species, and by creating nursery rhymes concerning the traditional use of selected species.

### 2.3. Students from 4th to 7th Grade (N. of Participants = 112)

The total number of species mentioned by the students via questionnaires was 41, belonging to 22 botanical families. The most recurrent families were Asteraceae (number of use reports = 102), Lamiaceae (n = 82), Theaceae (n = 56) and Rutaceae (n = 49), while the most recurrent species were *M. chamomilla* (n = 95), *C. sinensis* (n = 56), *Mentha* spp. (n = 41) and *C. limon* (n = 34). The most used parts of the plant were leaves (n = 159), followed by flowers/inflorescences/aerial parts (n = 155). Infusion was the most recurrent preparation (n = 234), followed by ‘other preparation’ (i.e., juice obtained from fruits such as lemon or orange, n = 88), and the plant used ‘as it is’ (n = 45). Oral administration was undoubtedly the most mentioned (n = 322), followed by external/topical (n = 49) and inhalations (n = 16).

Among the most recurrent categories of pathologies/apparatuses, we can find respiratory tract infections (n of use reports = 123; n of species = 26), followed by digestive problems (n = 91; n = 15) and nervous system (n = 67; n = 8). Further information, including ICFs for every category, can be found in Table 1.

By excluding the circulatory system (due to the low number of URs), the categories with higher agreement among the informants were nervous system disorders (ICF = 0.89), ophthalmic ailments (0.87), digestive tract disorders (0.84) and respiratory tract infections (0.80).

Specifically, for the nervous system, students mainly reported infusions of *M. chamomilla* and *C. sinensis*, mainly in their commercial forms, to facilitate sleeping. For ophthalmic ailments, they reported compresses of the infusion of *M. chamomilla* but also eating *Daucus carota* L. and *Vaccinium myrtillus* L. (bought at the market or cultivated) to maintain healthy eyesight. In the category of digestive problems, infusions of *M. chamomilla* and *C. sinensis* and the juice of *C. limon* were reported as useful remedies for tummy ache and nausea. *C. limon* was also considered digestive.

Ninety-six percent of the students cited at least 1 species, while 36% knew and reported more than 3 species each. Compared to our previous work [8], the students from Valle Imagna were notably more knowledgeable than the students from Tremezzina on medicinal species and uses (78% of the children with at least 1 species and 9% with more than 3 species each). This could depend on the older age of the students of Valle Imagna (8–14 vs. 6–10 in Tremezzina) and the types of questionnaires adapted by age, but also on the more rural territory that characterizes Valle Imagna.

However, similarly to the results obtained in Tremezzina, it is also important to highlight that around 40% of the use reports from the students of the Valle Imagna referred to exotic species or purchased plant material and thus that the uses could often be considered influenced by sources other than the traditions of the territory.

**Figure 1:**
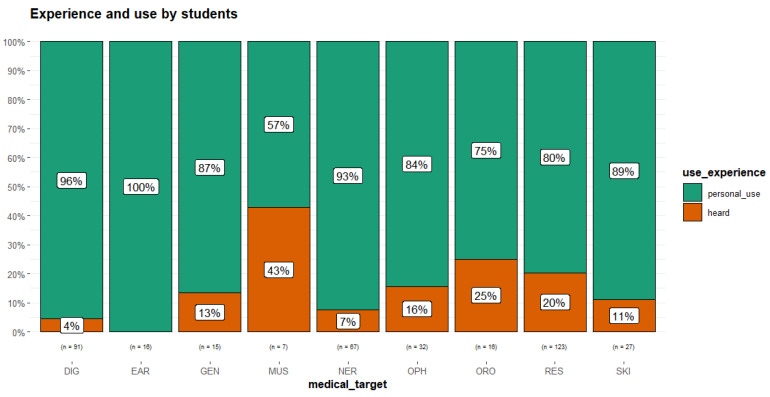
Experience of use reported by the students (medical_target considered only with number of URs (n) > 3). Digestive tract disorders (DIG); afflictions of the ear (EAR); general condition (GEN); musculoskeletal system disorders and traumas (MUS); nervous system disorders (NER); ophthalmic ailments (OPH); oropharyngeal cavity affections (ORO); respiratory tract infections (RES); skin diseases and traumas (SKI).

As can be observed in Figure 1, the experience of the use of plants in the categories of pathology was reported as mostly ‘personal’ by the students. This information is consistent with the fact that most of the remedies for common problems are usually administered to children by their parents or grandparents.

Further information on the species reported by the students can be found in Table S1 of the Supplementary Materials.

### 2.4. Parents’ Generation (N. of Participants = 96)

The number of species mentioned by the parents was 76, belonging to 38 botanical families. The most recurrent families were Lamiaceae (number of use reports = 119), Asteraceae (n = 107), Malvaceae (n = 64) and Lauraceae (n = 23), while the most recurrent species were *Malva sylvestris* L. (n = 49), *Mentha* spp. (n = 29), *M. chamomilla* (n = 27), *C. sinensis* (n = 56) and *Taraxacum* sect. *Taraxacum* (n = 27). The most used parts of the plant were leaves (n = 224), followed by flowers/inflorescences/aerial parts (n = 148) and hypogeal parts (n = 44). Infusion was the most recurrent preparation (n = 304), followed by ‘other preparation’ (n = 54) and decoction (n = 39). Oral administration was once again the most reported (n = 385), followed by topical (n = 75) and compress (n = 10). It is interesting to highlight that some of the preparations, specifically creams/ointments/macerated oils topically applied that were once personally prepared with spontaneous or cultivated species, were often replaced with commercial products containing the same herbal ingredient (i.e., *Calendula officinalis* L., *Hypericum perforatum* L., *Thymus vulgaris* L., etc.).

Among the most recurrent categories of pathologies/apparatuses, we can find digestive problems (n. of use reports = 98; n of species = 29), general condition (general anti-inflammatory, tonic, etc. n = 83; n = 34) and respiratory tract infections (n = 81; n = 23). Further information, including ICFs for every category, can be found in Table 2.

The categories with higher agreement among the informants were nervous system disorders (ICF = 0.78), respiratory tract infections (0.73), oropharyngeal cavity affections and skin diseases and traumas (0.72 each) and digestive tract disorders (0.71).

**Figure 2:**
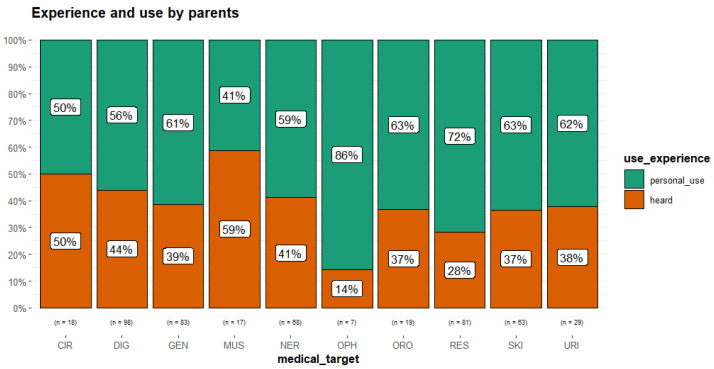
Experience of use reported by parents (medical_target considered only with number of URs (n) > 3). Circulatory system disorders (CIR); digestive tract disorders (DIG); general condition (GEN); musculoskeletal system disorders and traumas (MUS); nervous system disorders (NER); ophthalmic ailments (OPH); oropharyngeal cavity affections (ORO); respiratory tract infections (RES); skin diseases and traumas (SKI); urinary tract problems (URI).

For the parents, the experience of the use of plants was almost equally distributed in the categories of pathology between ‘personal’ and ‘heard’ (Figure 2).

Specifically, for the nervous system, the parents’ generation mainly reported infusions of *M. chamomilla*, *Tilia cordata* Mill., *Lavandula angustifolia* Mill. and *Melissa officinalis* L. as hypnotic-sedative and to facilitate sleep, while infusions of *L. angustifolia*, *M. officinalis* and *Mentha* spp. were also considered useful anxiolytic remedies. Infusions or essential oils (mainly in their commercial forms) of *L. angustifolia*, *Mentha* spp., *Ocimum basilicum* L. and *M. officinalis* were also used against headaches.

For the respiratory tract, inhalations using infusions of the aerial parts of *T. vulgaris*, *Sambucus nigra* L., *Taraxacum* sect. *Taraxacum* and *Salvia rosmarinus* Spenn. were administered for the treatment of cough, while in the case of common colds *Mentha* spp., *T. vulgaris*, *S. nigra* and *T. cordata* were employed. For sore throat, syrups of flowerhead of *Taraxacum* sect. *Taraxacum* were reported, as well as infusions of leaves *M. sylvestris* or of the bark of *Cinnamomum verum* K.Presl with *Citrus* x *aurantium* L. peels and decoctions of *Zingiber officinale* Roscoe.

For the oropharyngeal cavity, gingivitis and toothache were treated with infusions (taken orally or used as mouthwashes) of *M. sylvestris*, *S. rosmarinus* and *Salvia officinalis* L.

For the category of skin diseases and traumas, leaf gel of *Aloe vera* (L.) Burm.f., macerated oil of *C. officinalis* and of *H. perforatum*, and slices of hypogeal parts of *Solanum tuberosum* L. were applied on sunburns and burns, and irritated skin. Compresses of infusions of *Agrimonia eupatoria* L. and *M. sylvestris* and the macerated oil of *H. perforatum* were considered anti-inflammatory and wound-healing remedies.

For digestive tract problems, *Laurus nobilis* L., *Mentha* spp. and *Foeniculum vulgare* Mill. were considered digestive and eupeptic species; infusions of *M. sylvestris* or *S. nigra*, the juice of *A. vera* and the false fruits of *Rosa canina* L. taken orally were all reported as laxative remedies. Against stomachache, parents reported infusions of leaves from *T. vulgaris*, hypogeal parts of *S. tuberosum* boiled and eaten, and decoctions or infusions of *Laurus nobilis* L. and *Helichrysum italicum* (Roth) G.Don. Fruits of *F. vulgare*, leaves of *S. rosmarinus* and leaves of *Mentha* spp. in infusion were reported as carminative, while leaf gel of *A. vera* or infusions of leaves and flowers of *M. sylvestris* against gastritis and reflux. Against tummy ache, the parents reported infusions of *M. chamomilla*, *Origanum majorana* L. and *T. vulgaris.* Infusions of flowers of *S. nigra* or of leaves of *Cichorium intybus* L., A.v (L.) Burm.f. were reported as anti-inflammatory remedies for the intestine, while the decoction of leaves or roots of *Taraxacum* sect. *Taraxacum* and raw or cooked flowerheads of *Cynara cardunculus* L. were used as depurative for the liver.

Further information on the species reported by the parents’ generation can be found in Table S2 of the Supplementary Materials.

### 2.5. Grandparents’ Generation (N. of Participants = 35)

The number of species mentioned by the grandparents was 52, belonging to 28 botanical families. The most recurrent families were Asteraceae (number of use reports = 47), Lamiaceae (n = 40), Malvaceae (n = 30) and Rosaceae (n = 10), while the most recurrent species were *M. sylvestris* (n = 22), *Taraxacum* sect. *Taraxacum* (n = 13), *S. officinalis* (n = 10) and *M. chamomilla* (n = 9). The most used parts of the plant were leaves (n = 90), followed by flowers/inflorescences/aerial parts (n = 68) and fruits/infructescences/false fruits (n = 12). Infusion was once again the most recurrent preparation (n = 108), followed by ‘other preparation’ (n = 25) and decoction (n = 20). Oral administration was the most reported (n = 143), followed by topical (n = 32).

Among the most recurrent categories of pathologies/apparatuses, we can find general condition (number of use reports = 45; n of species = 19), digestive tract disorders (n = 34; n = 25) and respiratory tract infections (n = 30; n = 13). Further information, including ICFs for every category, can be found in Table 3.

By excluding external parasites (due to the low number of URs), the categories with higher agreement among the informants were general condition and respiratory tract infections (0.59 each), followed by nervous system disorders (ICF = 0.56) and skin diseases and traumas (0.55). It is interesting to highlight that these ICFs are notably lower than the ones calculated for children and lower than the ones for parents. These differences may stem from the distinct ways in which respondents across groups perceive emic categories (e.g., different ailments). For this reason, as Leonti and Wreckle note, the ICF does not necessarily capture the distribution of use reports within or between groups and therefore cannot always be considered a fully reliable tool for assessing the cultural significance of taxa [13].

**Figure 3:**
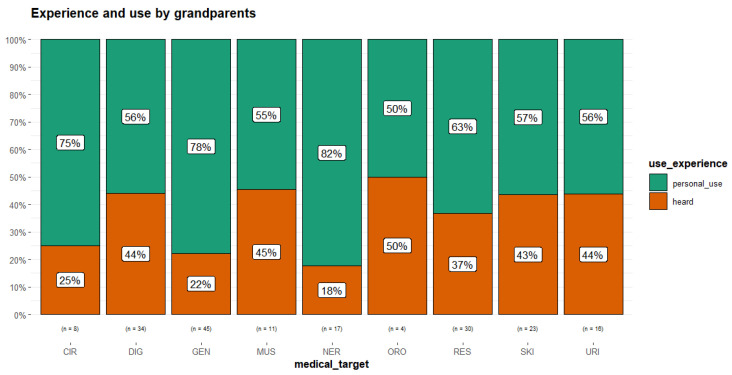
Experience of use reported by grandparents (medical_target considered only with number of URs (n) > 3). Circulatory system disorders (CIR); digestive tract disorders (DIG); general condition (GEN); muscoskeletal system disorders and traumas (MUS); nervous system disorders (NER); oropharyngeal cavity affections (ORO); respiratory tract infections (RES); skin diseases and traumas (SKI); urinary tract problems (URI).

Personal experience of use was mostly found in this generation, which was especially evident for some of the categories of pathology, such as nervous system, general condition and circulatory system (Figure 3).

Specifically, for the category general condition, infusions of *M. sylvestris*, *S. officinalis*, *Urtica dioica* L. and *L. angustifolia* were considered general anti-inflammatory for the whole body. General depurative remedies were the decoction of leaves from *Taraxacum* sect. *Taraxacum*, *C. intybus* and *T. vulgaris*. The leaves of *S. officinalis* (often in infusions) were reported as tonic and restorative, while rhyzomes of *Z. officinale* as immunomodulant.

For the respiratory tract, infusions of the aerial parts of *T. vulgaris*, *S. nigra* and *T. cordata* were administered for the treatment of cough, while in the case of common colds not only *T. vulgaris* and *T. cordata* were once again employed in infusion, but the false fruits of *R. canina* (eaten as they are or as jams) were taken.

For the nervous system, infusions of *M. chamomilla*, *S. officinalis* and *L. angustifolia* were used as hypnotic-sedative and to facilitate sleep, while infusions of *M. officinalis*, *M. chamomilla* and *Valeriana officinalis* L. were considered useful anxiolytic remedies. The hydroalcoholic macerate of *H. perforatum* was reported as anti-depressant.

Finally, for the category of skin diseases and traumas, the macerated oil of *C. officinalis*, as well as the compresses of *A. eupatoria*, *M. sylvestris* or *L. angustifolia* were considered anti-inflammatory and emollient. Once again, leaf gel of *A. vera* and macerated oil of *H. perforatum* were applied on sunburns and burns. Compresses of infusion of *Agrimonia eupatoria* L. were used as anti-inflammatory and wound healing. In the same fashion, the essential oil of *Melaleuca alternifolia* (Maiden and Betche) Cheel (the commercial form was diffusely known in the valley) was applied of wounds.

Further information on the species reported by the parents’ generation can be found in Table S3 of the Supplementary Materials.

### 2.6. Comparison Among Generations

**Figure 4:**
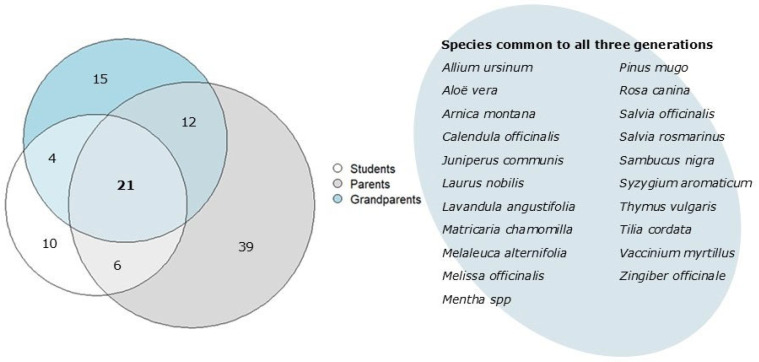
Venn diagram of the number of species reported by each generation.

As shown before, 41 plant species were reported by the students’ generation, 76 by the parents and 52 by the grandparents. Ten species were reported only by the children (*C. sinensis*, *C. annuum*, *C. aurantium*, *C. limon*, *C. arabica*, *C. avellana*, *N. tabacum*, *P. sylvestris*, *R. nigrum*, *T. cacao*), but among these only *C. avellana* is a spontaneous species of the territory. Aside from some isolated reports (i.e., *C. annuum*, *N. tabacum* and *R. nigrum*, with 1 informant and 1 UR each), children’s knowledge of these species actually came from their families, so they usually described remedies they were administered by their parents or grandparents. However, the fact that parents and grandparents did not mention them could be explained by how different generations would perceive something as ‘remedy’, ‘herbal remedy’, ‘plant remedy’. If as many as 40 children considered freshly squeezed orange and lemon juice enough of a remedy because “it’s rich in vitamin C!” or immediately thought of coffee as a stimulant and tonic drink, adults tended to think first of somewhat more complex remedies (decoctions, syrups, etc.) and disregard the simplest ones. On the other hand, 21 species were reported by all three generations (Figure 4). Out of these, only 9 species (*A. vera*, *A. montana*, *L. angustifolia*, *M. chamomilla*, *Mentha* spp., *S. rosmarinus*, *S. nigra*, *T. vulgaris* and *T. cordata*) were cited by at least 3 informants in each generation, reaching some kind of consensus. Considering that 31 species were cited just once in all the pool of informants, the number of singletons (species with isolated UR) we obtained were almost 3.5 times the ones that reached consensus among generations.

Specifically, other than being the most cited species, *M. chamomilla* was also reported by the informants for the same uses: in fact, the infusion of the flowerheads was always used for nervous system disorders (sedative hypnotic) and for digestive system problems (stomachache and abdominal pain). Although the species can be commonly found spontaneously in the territory, the plant material cited was commercially acquired most of the time, especially the one reported by the students.

Spontaneous species that were known by all three generations for the same uses were *C. officinalis* (compresses of the infusions used on the skin in case of inflammation), *M. officinalis* (infusion of the leaves for headaches), *Pinus mugo* Turra (syrup of the pinecones for colds, cough, sore throat) and *T. cordata* (infusions or syrups of the flowers for cough).

*Allium ursinum* L. and *Juniperus communis* L., common throughout the valley, though cited by all generations, were used differently: *A. ursinum* was known as a natural antibiotic agent by parents and children but was mentioned as a vermifuge and cholesterol-lowering remedy by the grandparents; *J. communis* was used by grandparents and parents to treat contusions and to improve circulation, while it was reported by children as useful against sore throat. This difference between the uses could be at least partly explained by considering the different health problems and needs of the three generations. For example, a remedy administered to improve circulation or to lower cholesterol is hardly useful to a child.

Cultivated or purchased species that are still linked to local traditions were *Syzygium aromaticum* (L.) Merr. & L.M.Perry and *A. vera*: the former to treat gingivitis and toothache, the latter for skin traumas, such as wounds and burns. The reported uses of both were the same in all three generations. Another example of non-native species, this time with more recent uses, was *Z. officinale* reported by all the generations as useful against colds or even as an immunostimulant.

We conducted further analysis of the familial units in order to verify the transmission of knowledge on the same species and uses along the three generations in the same unit. *S. nigra* was reported by every participant of the same unit. However, only grandparents and parents used the species as a remedy to treat the same category of pathology (respiratory tract) and specific pathology (cough), using the same part of the plant (flowers/inflorescences), while the child reported the same species for a different use, namely the leaves applied fresh against skin traumas (burns, sunburn). From the meetings with the students, it seems that some of the children still reported the use of leaves from different plant species (in particular trees) as ‘natural plasters’ when they get hurt while playing in the open air. This difference in the use of *S. nigra* between adults and children may thus simply reflect a different therapeutic need among generations, as well as and an empiricist and practical approach by the children, whereas the use of syrups or infusions of elderflowers is considered known by adults due to traditional use well established both on the territory of Valle Imagna [12] and in other areas of Lombardy [14,15] and Italy [16].

Among the 12 species (Figure 4) cited only by the adults’ generations, the case of *A. eupatoria* can be considered emblematic: infusions of the leaves were reported as useful anti-inflammatory and wound-healing agents by some of the grandparents and, to an even lesser extent, by some parents. None of the children reported the use of the species nor did they know the species itself. We have already analyzed the case of agrimony in Valle Imagna in previous works [12,17] and we highlighted that for this species, as well as for other ones, historical events (i.e., extensive emigrations from the valley during the early second half of the 20th century) may have played a role in the loss of traditional knowledge linked to the their uses. If there are very few elderly people that still use or even remember the species, the fact the even fewer adults and none of the children learned the traditions concerning this species is only to be expected and this consideration could be extended also to other plants.

In the case of other species, such as *C. intybus* used by the adults as digestive, laxative, depurative or *Crataegus monogyna* Jacq. used as hypotensive, the difference of therapeutic needs between adults and children could be taken into account. Considering that children reported mostly species and uses that they experienced themselves based on what the adults administered for common disorders (tummy ache, stomachache and nausea, relaxation, eye inflammation), it is clear that uses such as depurative or hypotensive are not common among children.

**Figure 5:**
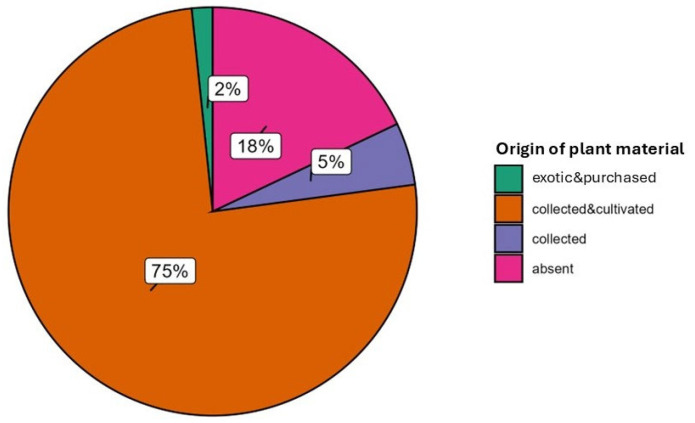
Origin of the plant material used by the informants.

**Figure 6:**
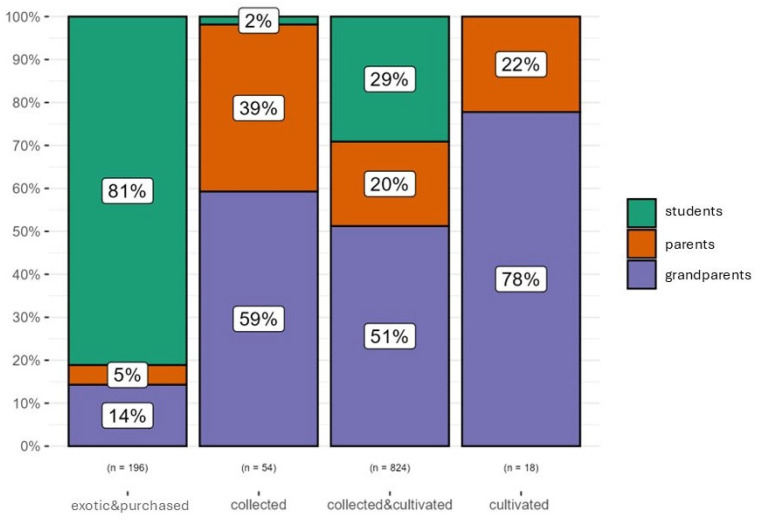
Origin of the plant material used by the informants per generation.

From Figure 5 and Figure 6, we can also observe the different origins of the plant species: in 82% of the uses, the reports referred to species that could be found both spontaneously and cultivated in the territory, while in 18% of the cases, the species were purchased (i.e., exotic species, such as *C. sinensis*, *Coffea arabica* L., *C. verum*, *M. alternifolia*, *Eucalyptus globulus* L., *S. aromaticum*, etc., but also *M. chamomilla*, which could be collected wild, cultivated in the garden or bought in the commercial form of sachets). *A. vera*, though not autochthonous to Valle Imagna, has long been cultivated in vases in the valley: almost in every household a vase of aloe could be found. The use of spontaneous species of the territory was more prominent among the adults while, as mentioned before, the citation of exotic species was widespread among the students (Figure 6).

As shown in Table 4, even considering the 10 most reported species in each generation it can be observed that children tended to give more importance to species not spontaneous in Valle Imagna, sometimes considering as ‘medicinal preparations’ even very common commercial products or common preparations (infusions) such as chamomile or tea obtained from sachets, lemon and orange juice, espresso coffee, etc. On the other hand, both parents and grandparents gave more importance to species that could be easily found in the territory (i.e., by mentioning *M. chamomilla* in its spontaneous *status* or species such as *S. nigra*, *T. cordata*, *J. communis*) and more often described more complex traditional preparations, such as syrups, ointments, macerated oils, though sometimes they also reported commercial products (essential oils, creams, etc.).

**Figure 7:**
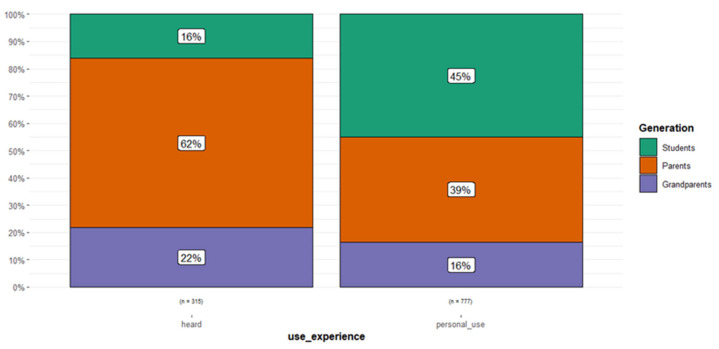
Experience of use reported by the informants per generation.

As for the experience of use, we pointed out that the majority of children reported their experience as ‘personal’. In Figure 7, we can observe that 84% of the uses labelled as only ‘heard’ (number of use reports = 315) were attributable to the adults’ generations (62% to the parents), while 45% of the uses considered ‘personal’ (n = 777) came from the children. As highlighted before, this could be the result of a number of factors, but it is probable that the children gave more weight to the remedies that the adults used to directly administer them, while parents and grandparents were especially prone to remembering also uses that they learned from the elders, but that they never put into practice.

**Figure 8:**
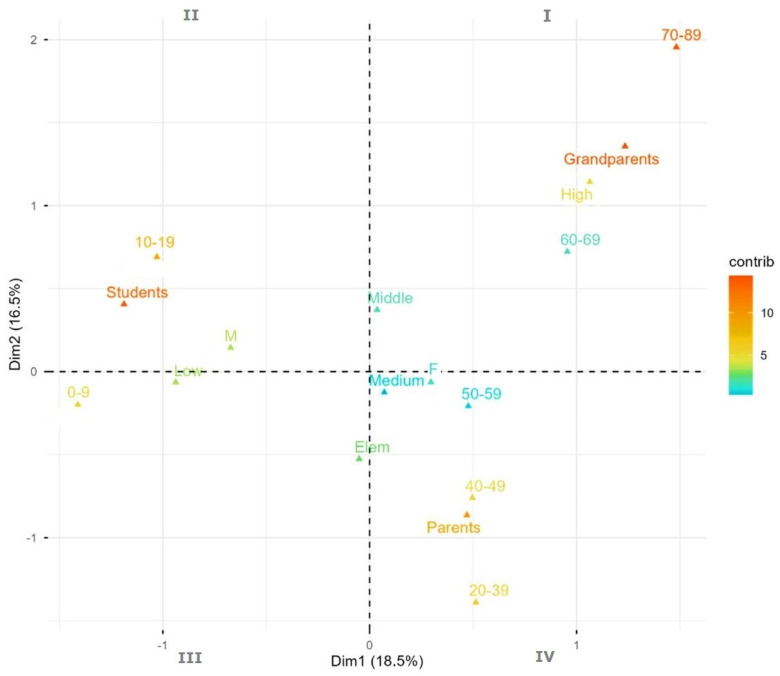
MCA Biplot (Dimensions 1 and 2). The plot shows the association between variable categories. Points close to each other are frequently co-occurring in the data. Multiple correspondence analysis biplot of selected variables (‘age_group’, ‘gender’, ‘level_veg_know’, ‘school_level’, ‘generation’) of first dimension (18.5% variance explained) and second one (16.5%). Grandparents, parents, students = the three generations considered in the study; high, medium, low = level of knowledge about plants; 0–9, 10–19, etc. = age range of informants; F, M = female, male genders of informants; elem, middle = elementary, middle schools, school grade. I, II, III, IV = quadrants.

The MCA analysis helped us analyze the insights of the data collected and effectively captured the underlying structure of how age, education level, generation and vegetable knowledge interrelated in our dataset. In Figure 8, Dimension 1 primarily represents the educational/age continuum, while Dimension 2 captures gender and knowledge-level variations within those groups. As can be observed, the biplot of the first two dimensions allows us to appreciate the existence of:

– Age–Knowledge Relationship: there is a clear gradient where younger students (0–9 in quadrant III, 10–19 in quadrant II) tend to have lower plant knowledge, adults (20–39, 40–49, 50–59 in quadrant IV) show medium knowledge levels, while older generations (60–69, 70–89, quadrant I) display more varied knowledge levels
– Gender Patterns: Dimension 2 reveals gender differences, with females clustered around medium knowledge levels (F, IV quadrant) and males showing proximity to low knowledge (M, II quadrant)
– Generation Clusters: students (II quadrant) are strongly associated with elementary school (Elem, III quadrant) and lower knowledge (Low, III quadrant), while parents (IV quadrant) are centrally positioned with medium knowledge (Med, IV quadrant) and grandparents (I quadrant) show more dispersion across knowledge levels
– Knowledge Progression: the analysis suggests that vegetable knowledge increases with age/educational level, from elementary to middle school and across generations.

If our previous work in schools [8] already highlighted strong consistency with the literature regarding the influence of age and gender on the traditional knowledge retained by the informants, many other works, some of them fairly recent, further confirmed the trend of older age and female gender correlated to deeper traditional knowledge [18,19,20].

Specifically regarding the gender, some authors found also a greater heterogeneity of the knowledge retained by men than women, suggesting a difference in the way information is spread within the same gender, with more communication among women [18].

Additionally, though this information cannot be specifically inferred from our data, some findings suggest also that women are the most responsible in the transmission of TEK to the children of a family or a community, even if the interest and time spent in these kinds of activities by children is less and less prominent by the day, not only in an urbanized setting, but also in a more rural one [21].

Though the influence of gender is sometimes visible from a qualitative point of view (the types of species and uses), rather than a quantitative (the number of species and uses), gender is still a variable that needs to be taken into account [19].

### 2.7. Weaknesses of the Methodological Approach and the Materials Used

From the work of analysis of the data collected during our investigation in the schools, we became aware of some weaknesses in the design of the study. We wish to explain them in this section of the work, with the aim of helping us and other researchers improve future attempts at this kind of research.

First, some of the materials and methods derived from Bruschi et al. 2019 [8] for the interviews should be enhanced or better adapted: the younger children (1st to 3rd grades) should be included in the semi-structured interviews via questionnaires because, despite their very young age, in this study they often proved to have even deeper knowledge on the topic than the older students (especially 3rd grade), greater willingness to involve themselves and their families, and simultaneously a certain rigor in collecting data. On the same subject, the questionnaires should not be differentiated based on age. In our previous work, the children involved in the study were only in primary school, so the simplified version was designed for the children and the more articulated one for the families. In the case of Valle Imagna, however, many students of 4th and 5th grades found the ‘adapted’ questionnaire more difficult to comprehend and compile than the one designed for older students and families. It would probably be more effective to maintain the same type of questionnaires for younger and older students, as well as the parents and grandparents, for example, by taking more time during the phase of compilation in class and by offering more support. This way, all data collected would be more homogeneous.

The questionnaires should also have a section (i.e., a specific column of the table) specifically dedicated to the source of every single use (the knowledge comes from family and from which member, society and/or books and web), in order to be able to thoroughly discuss the topic of the origin of the knowledge of every informant and every single species and use.

Finally, another hot topic is the level of involvement of the teachers in the study. While their help in our project was undoubtedly very important, the role of teachers should be more crucial and active in future, for they are the intermediary among us researchers (who are foreign to the territory), the children (whom they see every day) and the families (with whom they build a relationship of trust during their years at school). A more notable participation of the teachers could bring a higher degree of participation and interest by the students and, consequently, a higher degree of involvement by the families. Surely, the study was on a voluntary basis, but a greater boost from the teachers could have meant the collection of a higher number of complete questionnaires in grandparents’ and parents’ generations, which would have given us the ability to follow more easily the transmission along complete familiar units.

### 2.8. Value of the Project in Defining the Role of Ethnobotany

The concepts of involving the population of an investigated territory and then returning the knowledge collected during the field work back to the population of origin are part of the Citizen and the Open Science that abide perfectly by the characteristics of an ethnobotanical investigation, always so deeply rooted in a territory and its people. As we described in Giuliani et al. 2025 (Chapter 6, in course of publication [22]), an ethnobotanical investigation can be imagined divided into different steps—from the pre-field and field work, to the laboratory work, landing to the return to the population phase arranged in a cycle that begins and ends within the territory of origin.

The return to the population phase of an ethnobotanical survey is often seen as the final step in which the traditional knowledge of a territory is restored and given back to the whole population, as a means to stop erosion and help people become more aware of the value of their traditions. We abide by this phase too by publishing informative notebooks directed at the students and their families [23,24], but this is not the only way in which we complied.

Our work can be read in the key of the ethnobotanical survey that acquires a tangible social and cultural role already from the beginning of the field phase. In our project, the return to the population phase intertwined from the beginning with the field interviews.

**Figure 9:**
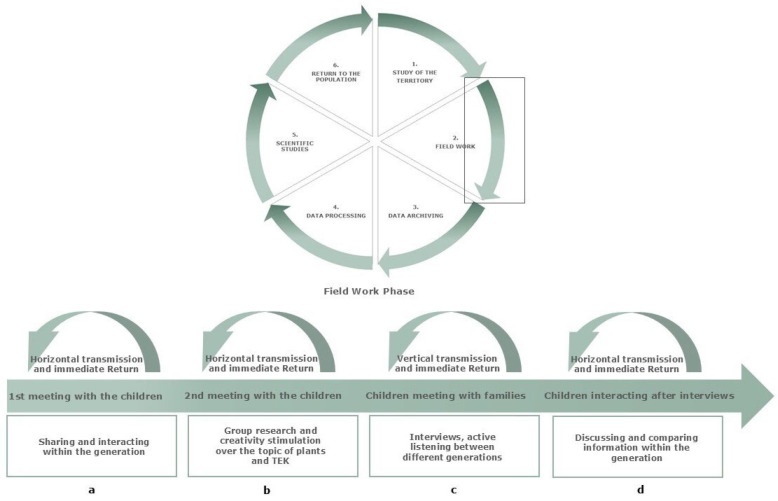
(**a**–**d**) Boomerang effect of every step in the field work phase on the return of knowledge to the children population and thus on the inversion of TEK erosion.

First of all, considering how the project developed, we were able to witness firsthand how, already during the first and second meeting with the students, horizontal transmission was facilitated within the children’s group. Under our guidance and with the encouragement of the teachers, students found themselves in the classroom sharing their knowledge and findings and interacting with each other. They were invited to do more research on plant species selected by them, to create nursery rhymes concerning the traditional uses of these species, to collect plant material and produce drawings (Figure 9a,b).

Moreover, during interviews, ethnobotanical researchers usually become ‘containers’ that are filled with traditional knowledge of a certain territory, they become mindful and aware of notions, traditions, anecdotes of the territory. By conferring the role of ethnobotanical researchers to the students, by giving them the responsibility of collecting data directly from the source (parents and grandparents), we also assigned to them the role of ‘containers’ of that traditional knowledge. This dialogue, which was mediated by the interviews (that we provided and illustrate to the children), became the means to acquire TEK/LEK and thus turned into the true moment of the return to the population (Figure 9c).

All people involved in the study were invited to the direct intergenerational exchange of information: the grandparents, in the first place, were invited to reprise their role of primary holders of traditional knowledge that cannot remain in the hands of the older generations alone, knowledge that must overcome mnemonic and communicative barriers and be passed down to their children and grandchildren. On the other hand, children were invited to spend their time attentively listening to the raw primary data, to accept the role of new ‘containers’ of TEK of their own territory. Thus, the ethnobotanical survey not only becomes a precious tool to foster valuable and beautiful social interactions among generations, but also the vertical transmission of knowledge to reverse, even only partially, the trend of erosion.

Finally, during final meetings in the classrooms, held after the interviews, students were asked to address an audience of peers (their classmates) with their results. Horizontal sharing was involved once again (Figure 9d), but this time their knowledge was enriched by what emerged during the family meetings. In this way, the children’s ethnobotanical knowledge became more articulated compared to the first sessions. For example, while in the questionnaires most of the children mentioned that hot tea was a remedy that helped them fall asleep (as they associated a warm drink with comfort before bedtime), during the interviews with their families they learned that it was actually chamomile, not tea, that had a relaxing and sleep-inducing effect.

Both horizontal and vertical transmission are crucial dynamics to fight disinterest, plant blindness and TEK/LEK erosion in younger generations [1,25,26]. Not only this, but the active participation required by this type of project and its practical nature also facilitated learning by the children, as already established in the literature [11].

Thus, each moment of the field phase, from the first encounters with the children characterized by free listing of known plant species, to the moments of interviews between children and adults, to the exchange of anecdotal information among the children during the last encounters, created several different little instances of immediate and intrinsic return to the children population that could be described as a ‘**boomerang effect**’ (Figure 9a–d) and which became an immediate and intrinsic tool to fight and reverse the trend of TEK erosion.

## 3. Materials and Methods

### 3.1. Area of Investigation

**Figure 10:**
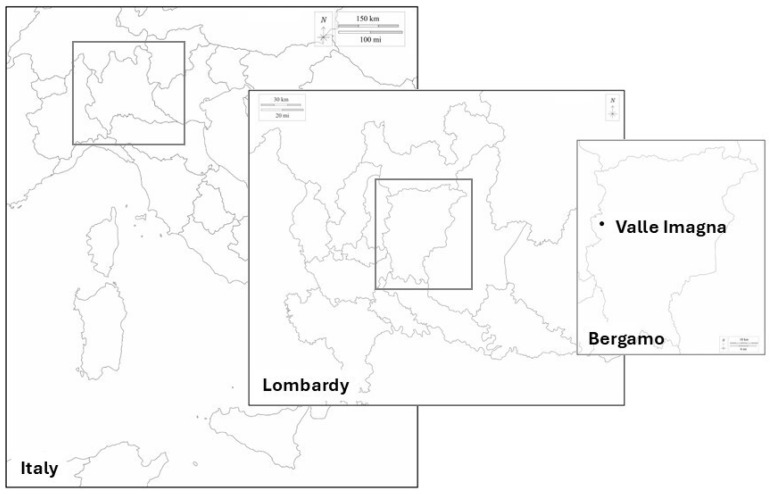
Valle Imagna (Province of Bergamo, Lombardy Region, North of Italy). Map obtained from the original maps at https://d-maps.com/m/europa/italia/italie_it/italie_it20.pdf (accessed on 20 January 2025), https://d-maps.com/m/europa/italia/lombardie/lombardie16.pdf (accessed on 20 January 2025), https://d-maps.com/m/europa/italia/bergamo/bergamo06.pdf (accessed on 20 January 2025).

Valle Imagna is a pre-alpine valley in the province of Bergamo (Western Orobic Alps, Lombardy, Northern Italy, see Figure 10) of 108.64 square kilometers, 70.89 of which belong to the upper Valle Imagna. The entire territory is considered ‘mountain’, even though some of the municipalities do not go over 500 m a.s.l. The valley is named after the Imagna river, which is the main body of water and stems from the foot of the highest mountain of the area (Mount Resegone, 1875 m a.s.l.). The weather is characterized by abundant rainfall and limited temperature excursions, which allow for the growth of abundant vegetation, mainly deciduous trees and herbaceous species typical of the pre-alpine territories [27]. We based our investigation on materials and methods described in Bruschi et al. 2019, with some needed adaptations that will be described throughout this section of the paper [8]. Thus, we focused first on the schools of upper Valle Imagna and the students, specifically the Comprehensive Institute of Sant’Omobono Terme (BG) in the primary school complexes of Corna Imagna (736 m a.s.l.), Mazzoleni (472 m a.s.l.), Rota d’Imagna (690 m a.s.l.) and Selino Basso (427 m a.s.l.) and the lower secondary school complex of Sant’Omobono Terme (427 m a.s.l.). In the second part of our investigation, we expanded the pool of informants involving the students’ parents and grandparents.

### 3.2. Grades and Number of Students

The study first involved students from different grades of both primary and lower secondary school. In Table 5, all grades and numbers of students in each class are described.

### 3.3. Outline of the Meetings in the School

From October 2022 to December 2024, the study unfolded through different phases, easily described as meetings. In Table 6, every meeting is outlined.

Specifically, after the introductory meeting with the Dean of the Institute (October 2022), in January 2023 the first meeting with the teaching staff took place, in order to explain the expected phases to the teachers, to describe to them the materials and methods and the degree of their involvement, to collect their voluntary adhesions to the project and to draft a first timetable with their help.

By the end of February and the beginning of March 2023, in every participant class we scheduled the first of two meetings. This two-hour meeting took place in the presence of at least two members of the research group and the teachers involved in every class. The aims of this first meeting were to describe the project to the students and optimize their participation and involvement. We fostered moments of exchange and debate on plant species of their territories in order to pique the interest of the students and convey the importance of their active participation in the study. We then proceeded to collect data on the known uses of plant species, with a focus on the medicinal sector of use. For the students of first to third grade, we used the methodology of free listing and conversation with the research members, who took notes on all the uses reported. For the classes of fourth to seventh grade, we described in detail the questionnaires outlined ad hoc for the structure interviews (described in the following section) and asked them to compile them on the spot, with the supervision and help of the researchers, when needed. The complete questionnaires were then collected and temporarily stored by the teachers.

Between the second half of March and the beginning of April 2023, we scheduled the second meeting with the students in order to collect all the products made by the students in between our meetings (drawings, presentations, anecdotes, notebooks, nursery rhymes) and to present and explain to them the questionnaires addressed to the parents and grandparents (described in the following section). From then on, the students were officially enrolled in the project not only as ‘subjects’ and ‘informants’ but also as ‘ethnobotanical researchers’. By the end of the meeting, four questionnaires were given to each student, with the task of interviewing their parents and grandparents. The participation of the relatives was maintained on a voluntary basis, so the students were given two weeks to collect as many questionnaires as possible, without any repercussions if they were not able to fulfil the task. During the following two weeks, the teachers collected all the questionnaires, dividing them into family units.

Between the first and second meeting with the students, we organized an evening meeting with their families, in an informal setting, in order to present the project and maximize their involvement.

Finally, in December 2024 we scheduled a final meeting in all the complexes, in order to deliver to the students and their families a token of the return to the population phase of the survey. This token took the shape of two divulgative notebooks containing some of the results of the investigation, along with the collected recipes, drawings, rhymes, etc. [23,24].

### 3.4. The Questionnaires

The questionnaires were based on the materials and methods described by [8] with some adaptations. With the presence of older students (11–13 years old), we decided to differentiate the questionnaires given to fourth and fifth graders from the one given to sixth and seventh graders, as described below.

The questionnaires for the primary school were designed to guide the students through compilation by means of explanatory drawings. It presented both closed and open questions subdivided into three sections. The first section was dedicated to anagraphical data. The second section, which was pictorial, concerned pathologies and remedies. As an example, we cite “When I cannot sleep, my parents/grandparents give me: A. pill; B. syrup; C. suppository; D. injection; E. drops”. The third section was the open one, in which the students were asked to write down the alternative plant remedies that they were given for the same problem. The considered disorders were earache, eye inflammations, stomach and tummy ache, vomit, wounds and burns, colds and cough, toothache, pain in the leg/foot/arm, insect bites, headache, sleep disorders.

The questionnaires for the lower secondary school were still articulated in three sections. The first section was dedicated to anagraphical data. In the second section, the students were asked to list the names of all the plants they knew, regardless of their traditional uses. The third section was a table subdivided into eight columns: plant name, treated disorder, part of the plant, preparation and administration modes, other plants used in the same remedy (if applicable), place and time of collection, current use/relevance, other notes. Specifically, for the column ‘current use/relevance’ there were four guided choices: 1. I still use this remedy; 2. I used it in the past; 3. I never used this remedy, I saw someone use it/someone told me, but it is still used nowadays; 4. I never used this remedy, I saw someone use it/someone told me and it is not used nowadays anymore.

The questionnaires for parents/grandparents were different from the ones for the lower secondary school only in the information asked for in the anagraphical section.

### 3.5. Data Archiving and Analysis

All data obtained from the questionnaires (from 4th grade to grandparents) was filed away in a database, organized in an Excel^TM^ spreadsheet (Microsoft, Redmond, WA, USA) where each row represented a ‘citation’ or ‘use report’ (UR), defined as “a single use reported for a single species by a single informant”. Every citation was considered ‘distinct’, when differing from one another in at least one of the following fields: species, informant id code, category of use, part of the plant, preparation and administration form. Data were subdivided into three sheets: students, parents and grandparents. Each informant was given a univocal code and information on the degrees of kinship among the informants were noted. Data was first processed and analyzed by means of Pivot tables.

The Informant Consensus Factor (ICF) was calculated according to Trotter and Logan (1986) as follows: ICF: (nur − nt)/(nur − 1), where nur is the number of use reports for each category of use (apparatus) and nt is the number of species used for each apparatus [28].

### 3.6. Statistical Analysis

A statistical analysis was conducted in two complementary phases to address the relationship between a determinate counting dependent variable (‘plant_species’), consisting of the number of species indicated by each subject participating in the questionnaire, and some key socio-demographic variables (age_group, gender, school_level). Also, a categorical derived variable ‘level_veg_know’ was obtained from ‘plant_species’ variables classifying one in three knowledge levels labeled ‘Low’ (0–2), ‘Medium’ (3–6) and ‘High’ (>6) corresponding to the first two thresholds of the 30th percentile and the 80th percentile, respectively. Initially, a Multiple Correspondence Analysis (MCA) was performed on the four key socio-demographic variables (‘age_group’, ‘gender’, ‘level_veg_know’, ‘school_level’) to reduce dimensionality and create a map of the associations between their different categories. The coordinates of the individuals on the first two principal dimensions from the MCA were plotted as a biplot thanks to the R FactoMineR package (version 2.12) [29]. Following this, a couple of Generalized Linear Models (GLM) were implemented with the variable ‘plant_species’ as the response variable. Given the nature of the response variable as a count, negative binomial distribution was specified and the ‘glm.nb’ function of the R MASS package was used [30]. The ‘level_veg_know’ variable was obviously excluded in predictors. The model included the original categorical variables—age_group, gender, school_level and generation—as fixed factors. This combined approach allowed for both an exploratory visualization of variable associations and a confirmatory test of their individual and collective influence on the botanical knowledge. The ‘age_group’ (‘0–9’, ‘10–20’, …, ‘70–89’) and ‘generation’ (‘Students’, ‘Parentes’ and ‘GranParents’) variables are confounders themselves so two GLM models were implemented excluding one of these. Model summaries and ANOVA tables of the models were provided by using R car package [31].

It is noteworthy that, considering that the groups in our study are not numerically balanced, we chose a General Linear Model (GLM) with Type III Sum of Squares for our Analysis of Variance (ANOVA), specifically designed to provide valid results even when cell sizes are unequal. Furthermore, the family of GLM used was not Gaussian but ‘negative binomial’ with a more appropriate glm.nb function. This approach tested each effect after adjusting for all other effects in the model, effectively ‘leveling’ the differences in group sizes and ensuring the robustness of our significance tests.

### 3.7. Plant Species

The scientific names of species and families were updated according to World Flora Online [32]. Exsiccata were collected for the spontaneous species of the territory and some of the home garden grown species and the specimens were given a code and stored in the Herbarium of the Agricultural Botany Laboratories of Florence (Erbario del Laboratori di Botanica Agraria di Firenze—FIAF).

## 4. Conclusions

In a context of erosion of traditional knowledge both in urbanized and in rural areas, globalization, disinterest and plant blindness shown by younger generations, we brought an example of an educative project in which children from primary and secondary schools were not only the subject of study of an ethnobotanical investigation aimed at taking a picture of the current situation of erosion, but also became directly involved in stopping and reverting the process.

From our results, it could be observed that higher levels of traditional knowledge were especially correlated with the older generations, while plants and uses reported by children were linked more to commercial preparations and exotic species. Traditional uses of the territory were rarely reported by children, whether for lack of interest in the topic, less time spent in the open or contamination from other sources. At the same time, historical events had chipped away at the traditional knowledge of the valley already among the older generations during the second half of 20th century, making it even more difficult to share it and pass it down to the younger ones.

One of the aims of our project was to instill in children greater interest in the natural environment and a desire to deepen knowledge and develop a new attitude towards the plant world, through reiterated contact with the topic, total involvement of the classes and fun. This form of massive involvement, also mediated by the teachers, through poetry, prose, workshops, experiments and drawings could be considered a first step to actively fight plant blindness among younger generations.

Additionally, the plant is not a sterile object of study, but it becomes a tool to enhance the relationship between plants and family; it is filled with family memories that have a stronger impact on the child. Such as it is, ethnobotany thus becomes an even more powerful tool than botany to teach the plant organism and the sciences related to the plant world to children. A project like the one we presented here is therefore potentially able to permeate school education with a proposal of novelty compared to classic programs and also allows one to stimulate the curiosity of the young generations towards their surrounding territory and the organism ‘plant’ while, at the same time, rooting traditional knowledge at a generational level and strengthening the ‘school–family–territory’ paradigm.

Where usually, through a project of this kind, from the older generations ethnobotanical knowledge is acquired, with the younger generations this knowledge can be built through the direct collaboration of ethnobotanical researchers with students, in concert with teachers and families. For example, before the interviews between the children and their families, *Agrimonia eupatoria* and its related traditional uses were hardly remembered by the older generations and never mentioned once by the younger ones.

Finally, for the ethnobotanical researchers themselves, these dynamics of continuous and organic exchange of roles and information, as well as the direct observation of the almost immediate effects that such a project can have, become extremely motivating and rewarding.

This is the crucial role of ethnobotany in such scenarios: we are aware that it is hardly possible to recover what is already lost, but our project represents tangible evidence of how ethnobotany could help preserve the remaining knowledge and identity of a territory and support the preservation already from the very first phases and with the complete involvement of the younger population.

## Figures and Tables

**Table 1:** Most reported categories of pathology (by number of URs and species), with related ICFs, in the group of students.

Category of Use (Apparatus)	Use Reports	Species	ICF
Circulatory system disorders (CIR)	2	1	1.00
Nervous system disorders (NER)	67	8	0.89
Ophthalmic ailments (OPH)	32	5	0.87
Digestive tract disorders (DIG)	91	15	0.84
Respiratory tract infections (RES)	123	26	0.80
Oropharyngeal cavity affections (ORO)	16	5	0.73
General condition (GEN)	15	6	0.64
Skin diseases and traumas (SKI)	27	11	0.62
Afflictions of the ear (EAR)	16	8	0.53
Musculoskeletal system disorders and traumas (MUS)	7	4	0.50
Fever (FEV)	3	3	0.00
External parasites (PAR)	1	1	

**Table 2:** Most reported categories of pathology (by number of URs and species), with related ICFs, in the group of parents.

Category of Use (Apparatus)	Use Reports	Species	ICF
Nervous system disorders (NER)	68	16	0.78
Respiratory tract infections (RES)	81	23	0.73
Oropharyngeal cavity affections (ORO)	19	6	0.72
Skin diseases and traumas (SKI)	62	18	0.72
Digestive tract disorders (DIG)	98	29	0.71
General condition (GEN)	83	34	0.60
Musculoskeletal system disorders and traumas (MUS)	17	8	0.56
Urinary tract problems (URI)	29	18	0.39
Other (OTH)	7	5	0.33
Ophthalmic ailments (OPH)	7	5	0.33
Circulatory system disorders (CIR)	18	14	0.24
Gynecological disorders (GYN)	3	3	0.00
Metabolic problems (MET)	2	2	0.00
Fever (FEV)	1	1	

**Table 3:** Most reported categories of pathology (by number of URs and species), with related ICFs, in the group of grandparents.

Category of Use (Apparatus)	Use Reports	Species	ICF
External parasites (PAR)	2	1	1.00
General condition (GEN)	45	19	0.59
Respiratory tract infections (RES)	30	13	0.59
Nervous system disorders (NER)	17	8	0.56
Skin diseases and traumas (SKI)	23	11	0.55
Musculoskeletal system disorders and traumas (MUS)	11	6	0.50
Urinary tract problems (URI)	16	11	0.33
Digestive tract disorders (DIG)	34	25	0.27
Circulatory system disorders (CIR)	8	8	0.00
Oropharyngeal cavity affections (ORO)	4	4	0.00
Ophthalmic ailments (OPH)	3	3	0.00
Other (OTH)	2	2	0.00
Gynecological disorders (GYN)	1	1	

**Table 4:** Ten species that received the highest number of reports (for each generation). Species in bold are common to all three generations.

10 Most Reported Species		
Children	Parents	Grandparents
***Matricaria chamomilla***	*Malva sylvestris*	*Malva sylvestris*
*Camellia sinensis*	***Mentha* spp.**	*Taraxacum sect. Taraxacum*
***Mentha* spp.**	***Matricaria chamomilla***	*Salvia officinalis*
*Citrus x limon*	*Taraxacum sect. Taraxacum*	***Matricaria chamomilla***
*Daucus carota*	*Lavandula angustifolia*	***Thymus vulgaris***
*Citrus x aurantium*	*Aloe vera*	*Urtica dioica*
***Thymus vulgaris***	*Sambucus nigra*	*Sambucus nigra*
*Lavandula angustifolia*	*Salvia rosmarinus*	*Tilia cordata*
*Coffea arabica*	***Thymus vulgaris***	*Juniperus communis*
*Aloe vera*	*Laurus nobilis*	***Mentha* spp.**

**Table 5:** Outline of the classes involved in the study, with number of students for each class.

Primary School		
School Complex	Grade	N. of Students
Corna Imagna	Multiage class 1st and 3rd	14
	2nd	8
	Multiage class 4th and 5th	19
Selino Basso	1st	24
	2nd	13
	3rd (A/B)	26
	4th (A/B)	38
Rota Imagna	Multiage class 1st and 2nd	10
Mazzoleni	3rd	16
	4th	12
Total students Primary school		180
Lower Secondary school (middle school)		
School complex	Grade	N. of students
Sant’Omobono (Selino Basso)	6th (A)	20
	6th (B)	20
	7th (B)	22
	7th (E)	20
Total students Lower Secondary School		82

**Table 6:** Schedule of the meetings of the study.

Meetings Schedule	
Introductory meeting with the principal	October 2022
Introductory meeting with the teachers	January 2023
First set of meetings with the students	January–March 2023
Evening gathering with the families	March 2023
Second set of meetings with the students	March–April 2023
Closing meeting with delivery of informative materials as outcome of the study	December 2024

## Data Availability

The original contributions presented in this study are included in the article/Supplementary Materials. Further inquiries can be directed to the corresponding author.

## Supplementary Materials

The following supporting information can be downloaded at: https://www.mdpi.com/article/10.3390/plants14223477/s1, Table S1: species and uses reported by the children in Valle Imagna (4th–7th grade). Circulatory system disorders (CIR); digestive tract disorders (DIG); afflictions of the ear (EAR); fever (FEV); general condition (GEN); gynecological disorders (GYN); metabolic problems (MET); musculoskeletal system disorders and traumas (MUS); nervous system disorders (NER); ophthalmic ailments (OPH); oropharyngeal cavity affections (ORO); Other (OTH); external parasites (PAR); respiratory tract infections (RES); skin diseases and traumas (SKI); urinary tract problems (URI). FIAF Code: official code of the Herbarium of the Agricultural Botany Laboratories of Florence; Table S2: species and uses reported by the parents’ generation. Circulatory system disorders (CIR); digestive tract disorders (DIG); afflictions of the ear (EAR); fever (FEV); general condition (GEN); gynecological disorders (GYN); metabolic problems (MET); musculoskeletal system disorders and traumas (MUS); nervous system disorders (NER); ophthalmic ailments (OPH); oropharyngeal cavity affections (ORO); Other (OTH); external parasites (PAR); respiratory tract infections (RES); skin diseases and traumas (SKI); urinary tract problems (URI). FIAF Code: official code of the Herbarium of the Agricultural Botany Laboratories of Florence; Table S3: species and uses reported by the grandparents’ generation. Circulatory system disorders (CIR); digestive tract disorders (DIG); afflictions of the ear (EAR); fever (FEV); general condition (GEN); gynecological disorders (GYN); metabolic problems (MET); musculoskeletal system disorders and traumas (MUS); nervous system disorders (NER); ophthalmic ailments (OPH); oropharyngeal cavity affections (ORO); Other (OTH); external parasites (PAR); respiratory tract infections (RES); skin diseases and traumas (SKI); urinary tract problems (URI). FIAF Code: official code of the Herbarium of the Agricultural Botany Laboratories of Florence.

## Abbreviations

The following abbreviations are used in this manuscript:

CIR	Circulatory system disorders
DIG	Digestive tract disorders
EAR	Afflictions of the ear
FEV	Fever
GEN	General condition
GYN	Gynecological disorders
ICF	Informant Consensus Factor
LEK	Local Ecological Knowledge
MET	Metabolic problems
MUS	Musculoskeletal system disorders and traumas
NER	Nervous system disorders
OPH	Ophthalmic ailments
ORO	Oropharyngeal cavity affections
OTH	Other
PAR	External parasites
RES	Respiratory tract infections
SKI	Skin diseases and traumas
TEK	Traditional Ecological Knowledge
URI	Urinary tract problems

## References

[1] Porcher V., Carrière S.M., Gallois S., Randriambanona H., Rafidison V.M., Reyes-García V. (2022). Growing up in the Betsileo landscape: Children’s wild edible plants knowledge in Madagascar. PLoS ONE.

[2] Ramirez C.R. (2007). Ethnobotany and the loss of traditional knowledge in the 21st century. Ethnobot. Res. Appl..

[3] Díez J., Meñika A., Sanz-Azkue I., Ortuzar A. (2018). Urban and Rural Children’s Knowledge on Biodiversity in Bizkaia: Tree Identification Skills and Animal and Plant Listing. Int. J. Humanit. Soc. Sci..

[4] Zarger R.K., Stepp J.R. (2004). Persistence of botanical knowledge among Tzeltal Maya children. Curr. Anthropol..

[5] Gallois S., Duda R., Reyes-García V. (2017). Local Ecological Knowledge among Baka Children: A Case of Children’s Culture?. J. Ethnobiol..

[6] Sousa D.C.P., Ferreira Júnior W.S., Albuquerque U.P. (2022). Short-term temporal analysis and children’s knowledge of the composition of important medicinal plants: The structural core hypothesis. J. Ethnobiol. Ethnomed..

[7] Vandebroek I., Balick M.J. (2012). Globalization and loss of plant knowledge: Challenging the paradigm. PLoS ONE.

[8] Bruschi P., Sugni M., Moretti A., Signorini M.A., Fico G. (2019). Children’s versus adult’s knowledge of medicinal plants: An ethnobotanical study in Tremezzina (Como, Lombardy, Italy). Rev. Bras. Farmacogn..

[9] Mattalia G., Svanberg I., Ståhlberg S., Kuznetsova N., Prūse B., Kolosova V., Aziz M.A., Kalle R., Sõukand R. (2023). Outdoor activities foster local plant knowledge in Karelia, NE Europe. Sci. Rep..

[10] Pany P. (2014). Students’ interest in useful plants: A potential key to counteract plant blindness. Plant Sci. Bull..

[11] Grasser S., Schunko C., Vogl C.R. (2016). Children as ethnobotanists: Methods and local impact of a participatory research project with children on wild plant gathering in the Grosses Walsertal Biosphere Reserve, Austria. J. Ethnobiol. Ethnomed..

[12] Milani F., Bottoni M., Bardelli L., Colombo L., Colombo P.S., Bruschi P., Giuliani C., Fico G. (2023). Remnants from the Past: From an 18th Century Manuscript to 21st Century Ethnobotany in Valle Imagna (Bergamo, Italy). Plants.

[13] Leonti M., Weckerle C.S. (2015). Quantitative and comparative methods in ethnopharmacology. Ethnopharmacology.

[14] Bottoni M., Milani F., Colombo L., Nallio K., Colombo P.S., Giuliani C., Bruschi P., Fico G. (2020). Using Medicinal Plants in Valmalenco (Italian Alps): From Tradition to Scientific Approaches. Molecules.

[15] Dei Cas L., Pugni F., Fico G. (2015). Tradition of use on medicinal species in Valfurva (Sondrio, Italy). J. Ethnopharmacol..

[16] Guarrera P.M. (2005). Traditional phytotherapy in Central Italy (Marche, Abruzzo, and Latium). Fitoterapia.

[17] Milani F., Muratore C., Biella S., Bottoni M., Rossi E., Colombo L., Colombo P.S., Bruschi P., Papini A., Landini P. (2025). From Traditional Medicine to the Laboratory: A Multidisciplinary Investigation on *Agrimonia eupatoria* L. Collected in Valle Imagna (BG, North of Italy). Plants.

[18] da Costa F.V., Guimarães M.F.M., Messias M.C.T.B. (2021). Gender differences in traditional knowledge of useful plants in a Brazilian community. PLoS ONE.

[19] da Silva Ribeiro Gomes C., Gama A.D.S., Cantalice A.S., da Mata P.T., da Silva T.C., de Medeiros P.M. (2024). Gender Influence on Local Botanical Knowledge about Medicinal Plants: A Study in Northeast Brazil. Ethnobot. Res. Appl..

[20] Müller J.G., Boubacar R., Guimbo I.D. (2015). The “How” and “Why” of Including Gender and Age in Ethnobotanical Research and Community-Based Resource Management. Ambio.

[21] Cruz García G.S. (2006). The mother—Child nexus. Knowledge and valuation of wild food plants in Wayanad, Western Ghats, India. J. Ethnobiol. Ethnomed..

[22] Giuliani C., Milani F., Bottoni M., Colombo L., Colombo P., Bruschi P., Fico G., Chandra S., Lata H. (2025). Ethnobotany of Medicinal Plants from High Mountains: The Case Study of Lombardy (Italy). High Altitude Medicinal Plants. Botany, Conservation and Cultivation.

[23] Fico G., Giuliani C., Milani F., Bottoni M., Colombo L., Maiellaro A. (2024). Quaderno Valle Imagna 2. Scuola & Tradizioni: Etnobotanica Tra i Banchi.

[24] Fico G., Giuliani C., Milani F., Bottoni M., Colombo L., Maiellareo A. (2024). Quaderno Valle Imagna 1. Scuola & Tradizioni: Etnobotanica Tra i Banchi.

[25] Reyes-García V., Broesch J., Calvet-Mir L., Fuentes-Peláez N., McDade T.W., Parsa S., Tanner S., Huanca T., Leonard W.R., Martínez-Rodríguez M.R. (2009). Cultural transmission of ethnobotanical knowledge and skills: An empirical analysis from an Amerindian society. Evol. Hum. Behav..

[26] Soldati G.T., Hanazaki N., Crivos M., Albuquerque U.P. (2015). Does environmental instability favor the production and horizontal transmission of knowledge regarding medicinal plants? A study in Southeast Brazil. PLoS ONE.

[27] Maconi G. (2006). Libri La Medicina Popolare in Valle Imagna. Componenti Magiche, Religiose ed Empiriche Tradizionali tra L’ottocento e il Novecento.

[28] Trotter R.T., Logan M.H., Etkin N.L. (1986). Informant consensus: A new approach for identifying potentially effective medicinalplants. Plants in Indigenous Medicine & Diet: Biobehavioral Approaches.

[29] Lê S., Josse J., Husson F. (2008). FactoMineR: An R package for multivariate analysis. J. Stat. Softw..

[30] Venables W., Ripley B. (2002). Modern Applied Statistics with S.

[31] Fox J., Weisberg S. (2019). An R Companion to Applied Regression.

[32] World Flora Online (WFO). https://www.worldfloraonline.org/.

