# Supplementary material for: Counteracting Traditional Knowledge Erosion: An Ethnobotanical Survey in Valle Imagna (Bergamo, Italy) to Foster Intergenerational Transfer
Source: Plants (Basel). 2025 Nov 14;14(22):3477. doi: 10.3390/plants14223477 (PMC12656025; doi:10.3390/plants14223477)
Supplement: Supplementary file 1 [file plants-14-03477-s001.zip › plants-3974510-supplementary.pdf]

plants

MDPI

Article

Counteracting Traditional Knowledge Erosion: an Ethnobotanical Survey in Valle Imagna (Bergamo, Italy) to Foster Intergenerational Transfer

Fabrizia Milani[1,2,*], Martina Bottoni[1,2], Alessia Maiellaro[1,2], Alfonso Crisci[3], Piero Bruschi[4], Claudia Giuliani[1,2], Gelsomina Fico[1,2].

[1] Department of Pharmaceutical Sciences, University of Milan, Via Luigi Mangiagalli 25, 20133 Milan, Italy. martina.bottoni@unimi.it; alessia.maiellaro@gmail.com; claudia.giuliani@unimi.it; gelsomina.fico@unimi.it

[2] Botanical Garden G.E. Ghirardi, Department of Pharmaceutical Sciences, University of Milan, Via Religione 25, 25088 Toscolano Maderno, Brescia, Italy.

[3] Institute of BioEconomy, National Research Council (CNR), Via Madonna del Piano 10, 50019 Sesto Fiorentino, Florence, Italy. alfonso.crisci@cnr.it

[4] Department of Agricultural, Environmental, Food and Forestry Science and Technology, University of Florence, Piazzale delle Cascine 18, 50144 Florence, Italy. piero.bruschi@unifi.it

[*] Corresponding author, fabrizia.milani@unimi.it

Supplementary Materials

Table S1: Species and uses reported by the children in Valle Imagna (4th-7th grade). Circulatory system disorders (CIR); Digestive tract disorders (DIG); Afflictions of the ear (EAR); Fever (FEV); General condition (GEN); Gynecological disorders (GYN); Metabolic problems (MET); Musculoskeletal system disorders and traumas (MUS); Nervous system disorders (NER); Ophthalmic ailments (OPH); Oropharyngeal cavity affections (ORO); Other (OTH); External parasites (PAR); Respiratory tract infections (RES); Skin diseases and traumas (SKI); Urinary tract problems (URI). FIAF Code: official code of the Herbarium of the Agricultural Botany Laboratories of Florence.

**Family/Species**<br>Code (FIAF)	**Part of plant**	**Category of pathology**
**Amaryllidaceae**		
Allium cepa L.	Hypogeal organs; Whole plant	SKI; MUS; RES
Allium ursinum L.<br>39153	Hypogeal organs	SKI; DIG; RES; GEN
**Apiaceae**		
Daucus carota L.	Hypogeal organs	EAR; EYE
Foeniculum vulgare Mill.	Fruits/Infructescences/False fruits	DIG

Asphodelaceae		
Aloë vera (L.) Burm.f.	Leaves	SKI
Asteraceae		
Achillea moschata Wulfen	Flowers/Inflorescences/Aerial parts	DIG
Arnica montana L. 39157	Flowers/Inflorescences/Aerial parts	MUS
Calendula officinalis L.	Flowers/Inflorescences/Aerial parts	SKI
Cynara cardunculus L.	Flowers/Inflorescences/Aerial parts	GEN
Matricaria chamomilla L. 39180	Flowers/Inflorescences/Aerial parts; Hypogeal organs	EAR; SKI; EYE; DIG; NER; RES; NER
**Betulaceae**		
Corylus avellana L. 39167	Leaves	CIR
**Cupressaceae**		
Juniperus communis L. 39177	Leaves	RES
**Ericaceae**		
Vaccinium myrtillus L. 39194	Fruits/Infructescences/False fruits	EYE; DIG
**Fabaceae**		
Glycyrrhiza glabra L.	Hypogeal organs	RES
**Grossulariaceae**		
Ribes nigrum L.	Fruits/Infructescences/False fruits	GEN
**Lamiaceae**		
Lavandula angustifolia Mill.	Flowers/Inflorescences/Aerial parts; Leaves	EAR; SKI; NER; RES; PAR; NER
Melissa officinalis L.	Leaves	DIG; NER; RES
Mentha spp	Flowers/Inflorescences/Aerial parts; Leaves	NER; ORO; EAR; DIG; RES; GEN

Salvia officinalis L.	Leaves	RES
Salvia rosmarinus Spenn.	Leaves	ORO; DIG; FEV; RES
Thymus vulgaris L.	Leaves	RES
**Lauraceae**		
Laurus nobilis L.	Leaves	SKI; DIG; RES
**Malvaceae**		
Malva sylvestris L.<br>39179	Flowers/Inflorescences/Aerial parts; Leaves	EAR; SKI; DIG; RES
Theobroma cacao L.	Seeds	NER; RES
Tilia cordata Mill.<br>39192	Flowers/Inflorescences/Aerial parts	NER; RES
**Myrtaceae**		
Eucalyptus globulus Labill.	Leaves	RES
Melaleuca alternifolia	Leaves	SKI
Syzygium aromaticum (L.) Merr. & L.M.Perry	Flowers/Inflorescences/Aerial parts	ORO
**Oleaceae**		
Olea europaea L.	Leaves	RES
**Pinaceae**		
Pinus mugo Turra	Leaves	MUS; RES
Pinus sylvestris L.	Leaves; Resin	RES; SKI
**Rosaceae**		
Rosa canina L.<br>39186	Flowers/Inflorescences/Aerial parts	RES
**Rubiaceae**		
Coffea arabica L.	Seeds	DIG; NER; GEN
**Rutaceae**		
Citrus x aurantium L.	Fruits/Infructescences/False fruits	EAR; DIG; RES; GEN

Citrus x limon (L.) Osbeck	Fruits/Infructescences/False fruits	EAR; DIG; RES
**Solanaceae**		
Capsicum annuum L.	Seeds	FEV
Nicotiana tabacum L.	Leaves	RES
Solanum tuberosum L.	Hypogeal organs	ORO; EYE; MUS
**Theaceae**		
Camellia sinensis (L.) Kuntze	Leaves ; Fruits/Infructescences/False fruits	ORO; EAR; EYE; DIG; NER; RES
**Viburnaceae**		
Sambucus nigra L. 39189	Flowers/Inflorescences/Aerial parts; Leaves	SKI; RES; SKI
**Zingiberaceae**		
Zingiber officinale Roscoe	Leaves; Hypogeal organs	RES; DIG; FEV

Table S2: Species and uses reported by the parents' generation. Circulatory system disorders (CIR); Digestive tract disorders (DIG); Afflictions of the ear (EAR); Fever (FEV); General condition (GEN); Gynecological disorders (GYN); Metabolic problems (MET); Musculoskeletal system disorders and traumas (MUS); Nervous system disorders (NER); Ophthalmic ailments (OPH); Oropharyngeal cavity affections (ORO); Other (OTH); External parasites (PAR); Respiratory tract infections (RES); Skin diseases and traumas (SKI); Urinary tract problems (URI). FIAF Code: official code of the Herbarium of the Agricultural Botany Laboratories of Florence.

**Family/Species** Code (FIAF)	**Part of plants**	**Category of pathology**
**Amaryllidaceae**		
Allium cepa L.	Hypogeal organs	RES
Allium schoenoprasum L. 39152	Whole plant	CIR
Allium ursinum L. 39153	Hypogeal organs; Whole plant	CIR; EYE; GYN; GEN
**Apiaceae**		
Daucus carota L.	Hypogeal organs	GEN
Foeniculum vulgare Mill.	Fruits/Infructescences/False fruits	DIG; EYE; CIR; URI; GEN

Petroselinum crispum (Mill.) Fus	Leaves	URI
**Apocynaceae**		
Vinca minor L.<br>39196	Leaves	RES
**Asparagaceae**		
Ruscus aculeatus L.<br>39188	NN	GEN
**Asphodelaceae**		
Aloë vera (L.) Burm.f.	Leaves; Latex or sap	SKI; DIG; GEN
**Asteraceae**		
Achillea millefolium L.<br>39150	Flowers/Inflorescences/Aerial parts	DIG; NER
Arctium lappa L.<br>39155	Leaves; Hypogeal organs	SKI; URI; GEN
Arnica montana L.<br>39157	Flowers/Inflorescences/Aerial parts	MUS; GEN
Artemisia absinthium L.<br>39158	Flowers/Inflorescences/Aerial parts; Leaves	DIG; URI
Calendula officinalis L.	Flowers/Inflorescences/Aerial parts	SKI; GEN
Centaurea benedicta (L.) L.	Whole plant	URI
Cichorium intybus L.<br>39165	Leaves; Hypogeal organs	GEN; DIG
Cynara cardunculus L.	Flowers/Inflorescences/Aerial parts	CIR; DIG
Echinacea purpurea (L.) Moench	Hypogeal organs	RES
Helichrysum italicum (Roth) G.Don<br>39173	Leaves	DIG
Hieracium pilosella L.<br>39174	Whole plant	CIR; URI; GEN

Matricaria chamomilla L.<br>39180	Flowers/Inflorescences/Aerial parts; Leaves	ORO; EYE; DIG; MUS; NER; GEN
Melissa officinalis L.	Leaves	NER
Taraxacum sect. *Taraxacum*<br>39191	Flowers/Inflorescences/Aerial parts; Leaves; Hypogeal organs; Whole plant	DIG; URI; RES; GEN; OTH
**Betulaceae**		
Betula pendula Roth<br>39161	Leaves	URI
**Boraginaceae**		
Pulmonaria officinalis L.<br>39184	Leaves	RES
**Brassicaceae**		
Capsella bursa-pastoris (L.) Medik. subsp. *bursa-pastoris*<br>39162	Fruits/Infructescences/False fruits	CIR
**Campanulaceae**		
Platycodon grandiflorus (Jacq.) A.DC.	Hypogeal organs	RES
**Cupressaceae**		
Juniperus communis L.<br>39177	Fruits/Infructescences/False fruits	CIR; MUS; GEN
**Ericaceae**		
Arctostaphylos uva-ursi (L.) Spreng<br>39156	Fruits/Infructescences/False fruits	URI
Vaccinium myrtillus L.<br>39194	Leaves ; Fruits/Infructescences/False fruits	CIR; GEN
**Fabaceae**		
Glycyrrhiza glabra L.	Hypogeal organs	SKI; CIR; URI; RES
Robinia pseudoacacia L.<br>39185	Flowers/Inflorescences/Aerial parts	DIG

**Fagaceae**		
Castanea sativa Mill.<br>39163	Leaves	RES
**Gentianaceae**		
Gentiana lutea L. subsp. lutea<br>39172	Hypogeal organs	DIG
**Geraniaceae**		
Pelargonium odoratissimum [Soland.]<br>39182	Leaves	CIR
**Hypericaceae**		
Hypericum perforatum L.<br>39175	Flowers/Inflorescences/Aerial parts	SKI; NER
**Juglandaceae**		
Juglans regia L.<br>39176	Leaves	SKI
**Lamiaceae**		
Lavandula angustifolia Mill.	Flowers/Inflorescences/Aerial parts; Leaves	SKI; URI; NER; RES; GEN
Melissa officinalis L.	Flowers/Inflorescences/Aerial parts; Leaves; Epigeal parts	NER; OPH; DIG; GEN
Mentha spp	Flowers/Inflorescences/Aerial parts; Leaves	DIG; ORO; MUS; NER; RES; GEN
Ocimum basilicum L.	Leaves	DIG; NER
Origanum majorana L.	Leaves	RES; GEN
Salvia officinalis L.	Leaves	ORO; DIG
Salvia rosmarinus Spenn.	Leaves; Woody stems/branches/twigs/shoots (suckers)	ORO; SKI; CIR; DIG; NER; FEV; RES; GEN
Teucrium chamaedrys L.	Unknown	DIG
Thymus vulgaris L.	Flowers/Inflorescences/Aerial parts; Leaves	RES; DIG; GEN

**Lauraceae**		
Cinnamomum verum J.Presl	Rhytidome	OPH; DIG; MET; RES; GEN
Laurus nobilis L.	Leaves	DIG; URI; MUS; NER; RES; GEN
**Malvaceae**		
Malva sylvestris L.<br>39179	Flowers/Inflorescences/Aerial parts; Leaves; Whole plant	ORO; GEN; SKI; EYE; OPH; DIG; URI; GYN; RES
Tilia cordata Mill.<br>39192	Flowers/Inflorescences/Aerial parts	NER; RES
**Moraceae**		
Ficus carica L.<br>39171	Woody stems/branches/twigs/shoots (suckers)	SKI
**Myrtaceae**		
Melaleuca alternifolia (Maiden & Betche) Cheel	Leaves; Whole plant	GYN; GEN; SKI
Myrtus communis L.	Leaves	RES
Syzygium aromaticum (L.) Merr. & L.M.Perry	Flowers/Inflorescences/Aerial parts	ORO
**Oleaceae**		
Olea europaea L.	Leaves	MET
**Papaveraceae**		
Chelidonium majus L.<br>39164	Latex or sap	SKI
Papaver rhoeas L.<br>39181	Flowers/Inflorescences/Aerial parts	NER; RES
**Pedaliaceae**		
Harpagophytum procumbens DC. ex Meisn.	Whole plant	MUS
**Pinaceae**		

Pinus mugo Turra	Fruits/Infructescences/False fruits ; Buds	RES
**Plantaginaceae**		
Plantago lanceolata L.<br>39183	Leaves	SKI; GEN
**Poaceae**		
Cymbopogon flexuosus (Nees ex Steud.) Will.Watson<br>39169	Leaves	NER; GEN
**Polygonaceae**		
Rheum rhabarbarum L.	Hypogeal organs	DIG
**Rosaceae**		
Agrimonia eupatoria L.	Flowers/Inflorescences/Aerial parts; Leaves; Whole plant	GEN; SKI
Crataegus monogyna Jacq.<br>39168	Flowers/Inflorescences/Aerial parts; Fruits/Infructescences/False fruits	CIR; NER
Rosa canina L.<br>39186	Fruits/Infructescences/False fruits; Flowers/Inflorescences/Aerial parts; Buds; Seeds	GEN; SKI; DIG; URI
Rubus idaeus L. subsp. *idaeus*<br>39187	Leaves	URI
**Rutaceae**		
Citrus bergamia Risso	Fruits/Infructescences/False fruits	DIG; URI; NER
**Solanaceae**		
Atropa belladonna L.<br>39159	Leaves	RES
Solanum tuberosum L.	Hypogeal organs	SKI; EYE; DIG; GEN
**Urticaceae**		
Urtica dioica L.<br>39193	Leaves	SKI; OPH; CIR; URI; MUS; GEN

**Valerianaceae**		
Valeriana officinalis L.<br>39195	Leaves; Hypogeal organs	NER
**Verbenaceae**		
Aloysia citrodora Paláu	Leaves	DIG; NER
**Viburnaceae**		
Sambucus nigra L.<br>39189	Flowers/Inflorescences/Aerial parts; Fruits/Infructescences/False fruits; Rhytidome	DIG; RES; GEN ; URI
**Zingiberaceae**		
Curcuma longa L.	Hypogeal organs	SKI; MUS; GEN
Zingiber officinale Roscoe	Hypogeal organs	DIG; RES

Table S3: Species and uses reported by the grandparents' generation. Circulatory system disorders (CIR); Digestive tract disorders (DIG); Afflictions of the ear (EAR); Fever (FEV); General condition (GEN); Gynecological disorders (GYN); Metabolic problems (MET); Musculoskeletal system disorders and traumas (MUS); Nervous system disorders (NER); Ophthalmic ailments (OPH); Oropharyngeal cavity affections (ORO); Other (OTH); External parasites (PAR); Respiratory tract infections (RES); Skin diseases and traumas (SKI); Urinary tract problems (URI). FIAF Code: official code of the Herbarium of the Agricultural Botany Laboratories of Florence.

**Family/Species**<br>Code (FIAF)	**Part of Plant**	**Category of use**
**Amaryllidaceae**		
Allium moschatum L.<br>39151	Hypogeal organs	CIR
Allium schoenoprasum L.<br>*39152*	Whole plant	CIR; DIG
Allium ursinum L.<br>39153	Leaves; Hypogeal organs	CIR; DIG
**Apiaceae**		
Foeniculum vulgare Mill.	Fruits/Infructescences/False fruits	CIR; DIG
Petroselinum crispum (Mill.) Fus	Leaves	URI

**Asphodelaceae**		
Aloë vera (L.) Burm.f.	Leaves	SKI; DIG
**Asteraceae**		
Achillea moschata Wulfen	Flowers/Inflorescences/Aerial parts	DIG
Arnica montana L.<br>39157	Flowers/Inflorescences/Aerial parts	SKI; MUS
Artemisia absinthium L.<br>39158	Flowers/Inflorescences/Aerial parts; Whole plant	DIG; URI
Bellis perennis L.<br>39160	Flowers/Inflorescences/Aerial parts	CIR
Calendula officinalis L.	Flowers/Inflorescences/Aerial parts	ORO; SKI; GEN
Cichorium intybus L.<br>39165	Flowers/Inflorescences/Aerial parts; Leaves; Whole plant	GEN; CIR; DIG; URI
Cirsium vulgare (Savi) Ten<br>39166	Leaves	SKI
Matricaria chamomilla L.<br>39180	Flowers/Inflorescences/Aerial parts; Leaves	DIG; NER; GEN
Taraxacum sect. *Taraxacum*<br>39191	Flowers/Inflorescences/Aerial parts; Leaves; Whole plant	DIG; URI; NER; RES; GEN
**Betulaceae**		
Betula pendula Roth<br>39161	Leaves	URI
**Brassicaceae**		
Brassica oleracea L.	Leaves	MUS
Lepidium sativum L. subsp. *sativum*	Leaves	GEN
**Caryophyllaceae**		
Lychnis flos-cuculi L. subsp. *flos-cuculi*<br>39178	Flowers/Inflorescences/Aerial parts	RES

**Cupressaceae**		
Juniperus communis L.<br>39177	Leaves; Fruits/Infructescences/False fruits	RES; CIR; DIG; URI; MUS
**Ericaceae**		
Vaccinium myrtillus L.<br>39194	Fruits/Infructescences/False fruits	DIG
**Fabaceae**		
Cassia senna L.	Whole plant	DIG
**Gentianaceae**		
Gentiana lutea L. subsp. *lutea*<br>39172	Leaves; Hypogeal organs	DIG
**Hypericaceae**		
Hypericum perforatum L.<br>39175	Flowers/Inflorescences/Aerial parts	URI; NER
**Lamiaceae**		
Lavandula angustifolia Mill.	Flowers/Inflorescences/Aerial parts	OPH; DIG; NER; GEN; SKI
Melissa officinalis L.	Leaves	DIG; NER
Mentha spp	Leaves	DIG; RES; GEN;
Ocimum basilicum L.	Leaves	DIG
Salvia officinalis L.	Leaves	ORO; OPH; DIG; GYN; NER; GEN
Salvia rosmarinus Spenn.	Leaves	CIR; DIG; RES; GEN;
Thymus vulgaris L.	Leaves	SKI; DIG; RES; GEN
**Lauraceae**		
Laurus nobilis L.	Leaves	DIG; GEN
**Linaceae**		

Linum usitatissimum L.	Seeds	DIG; GEN
**Malvaceae**		
Althaea officinalis L.<br>39154	Leaves	DIG
Malva sylvestris L.<br>39179	Flowers/Inflorescences/Aerial parts; Leaves; Epigeal parts	DIG; URI; RES; GEN; ORO; SKI; EYE; URI
Tilia cordata Mill.<br>39192	Flowers/Inflorescences/Aerial parts	RES; GEN
**Moraceae**		
Ficus carica L.<br>39171	Leaves	GEN
**Myrtaceae**		
Eucalyptus globulus Labill.	Leaves	RES
Melaleuca alternifolia (Maiden & Betche) Cheel	Leaves	SKI; PAR
**Myrtaceae**		
Syzygium aromaticum (L.) Merr. & L.M.Perry	Flowers/Inflorescences/Aerial parts	ORO; MUS
**Papaveraceae**		
Chelidonium majus L.<br>39164	Hypogeal organs	SKI
**Pinaceae**		
Pinus mugo Turra	Seeds	RES
**Rosaceae**		
Agrimonia eupatoria L.	Flowers/Inflorescences/Aerial parts; Leaves; Whole plant	SKI; EYE; URI; SKI
Crataegus monogyna Jacq.<br>39168	Flowers/Inflorescences/Aerial parts	GEN
Rosa canina L.<br>39186	Fruits/Infructescences/False fruits	URI; MUS; RES

**Rutaceae**		
Ruta graveolens L.	Leaves	EYE
**Solanaceae**		
Datura stramonium L.<br>39170	Leaves	RES
**Urticaceae**		
Urtica dioica L.<br>39193	Leaves; Hypogeal organs; Whole plant	URI; MUS; GEN
**Valerianaceae**		
Valeriana officinalis L.<br>39195	Leaves	NER
**Viburnaceae**		
Sambucus nigra L.<br>39189	Flowers/Inflorescences/Aerial parts; Fruits/Infructescences/False fruits	RES; GEN
**Zingiberaceae**		
Zingiber officinale Roscoe	Hypogeal organs	DIG; GEN
